# Systemic immune-inflammation index independently predicts poor survival of older adults with hip fracture: a prospective cohort study

**DOI:** 10.1186/s12877-021-02102-3

**Published:** 2021-03-04

**Authors:** Zhi-Cong Wang, Wei Jiang, Xi Chen, Ling Yang, Hong Wang, Yue-Hong Liu

**Affiliations:** Orthopaedic Center of Deyang City, Department of Orthopedics, People’s Hospital of Deyang City, Deyang City, 618000 Sichuan China

**Keywords:** Older adults, Hip fracture, Systemic immune-inflammation index, Prognosis, Mortality

## Abstract

**Background:**

The systemic immune-inflammation index (SII), based on peripheral platelet, neutrophil and lymphocyte counts, has been proven to be a promising prognostic indicator in various diseases. Hip fracture is a common injury among the older adults, and has become a global public health problem with high mortality and disability rates. However, the relationship between SII and the prognosis of hip fracture is not yet well-known. The aim of the this study was to explore the predictive value of SII in older adults with hip fracture undergoing surgery.

**Methods:**

This was a prospective cohort study performed from January 2014 to December 2018 at a orthopaedic center, China. The SII was calculated as platelet×neutrophil/lymphocyte counts. Univariable and multivariable Cox proportional hazard models were used to assess the association between SII and all-cause mortality.

**Results:**

A total of 290 older adults with hip fracture were included, and the mean (SD) age was 77.6 (8.6) years, and 189 (65.2%) were female. The median (IQR) SII was 759.4 (519.0–1128.7) × 10^9^/L. After a median follow-up time of 33.4 months, 13 (4.5%), 26 (9.0%) and 54 (18.6%) patients died within the 30-day, 1-year and last follow-up, respectively. Multivariable Cox analysis revealed that each increase of 100 units of SII was associated with a 8% increased hazard of death at 1-year follow-up (HR = 1.08, 95% CI: 1.01–1.17, *p* = 0.033), and 9% increased hazard of death at last follow-up (HR = 1.09, 95% CI: 1.03–1.15, *p* = 0.003).

**Conclusions:**

SII is associated with poor all-cause mortality in older adults with hip fracture undergoing surgery, and deserves further investigation and application in clinical practice.

## Background

With the rapid development of the aging population, hip fracture has become more and more frequent in the older adults. Globally, an estimated 1.6 million hip fractures occurred in 2000 [1], and the number may rise to 2.6 million in 2025 and 4.5 million in 2050 [2]. Despite improvements in perioperative care, it is well known that hip fracture is associated with substantial excess mortality in older adults, and the mortality rates range between 1.0 and 7.2% at 30 days, and significantly increase to 23.5% within 1 year after hip fracture [3–7].

In order to predict the prognosis of hip fracture, many assessment tools have been developed, such as Nottingham Hip Fracture Score (NHFS) [8, 9], Physiologic and Operative Severity Score for the enUmeration of Mortality and Morbidity (POSSUM) [10, 11], and Almelo Hip Fracture Score (AHFS) [12]. However, none of the existing tools showed excellent discrimination or calibration [11, 13]. Recently, some inflammatory markers, such as interleukin-6 (IL-6), C-reactive protein (CRP), prognostic nutritional index ratio (PNI), CRP/PNI ratio, and neutrophil-to-lymphocyte ratio (NLR) were found to be independently associated with increased early and long-term mortality after hip fracture [14–19].

The systemic immune-inflammation index (SII), which is a composite indicator integrating platelet, neutrophil and lymphocyte counts, has been proven to be a promising prognostic predictor in various diseases, including malignant tumors [20–22], coronary artery disease [23], acute ischemic stroke [24], and preterm premature rupture of the membranes [25]. Moreover, high SII (≥834.89) was newly identified as a good risk predictor for discriminating osteoporotic fracture risk in postmenopausal osteoporosis patients [26]. Recently, we also firstly reported that high platelet-to-lymphocyte ratio (PLR) was associated with increased 1 year all-cause mortality in older adults with hip fracture [27]. As far as we know, no studies have investigated the relationship between SII and mortality in older adults with hip fracture. Therefore, the aim of this study was to explore the predictive value of SII in older adults with hip fracture.

## Methods

### Study design and participants

This was a prospective cohort study using a hip fracture database from our orthopaedic center in Deyang city, China. As we previously described [27], patients were included in the database if the following criteria were met: (I) aged over 60 years; (II) diagnosed with hip fracture, not pathological fracture; (III) caused by a low-energy mechanism (defined as a fall from no greater than standing height); (IV) fresh fracture less than 3 weeks. After inclusion, we then entered the demographic details [age at admission (years), sex (male/female)], clinical characteristics [admission date and diagnosis, hip fracture type (femur neck fracture/intertrochanteric fracture), comorbidities, treatment (surgical/conservative), discharge date and diagnosis)], surgical information [date of surgery, type of surgery (internal fixation/arthroplasty), anaesthesia (general/spinal), and time to surgery], and laboratory data at admission (routine blood test, liver and kidney function, and electrolyte). In this study, patients with conservative treatment, no one follow-up information, chronic or acute infection within 48 h of admission, missing values for platelet, neutrophil, lymphocyte counts were excluded. The study was approved by the Institutional Ethics Committee of People’s Hospital of Deyang City.

### Assessment of SII

Whole blood samples (1.0 mL) were routinely obtained from all patients within 24 h after hospital admission, and blood routine examination was performed immediately by an automatic hematology analyzer (Sysmex XN2000, Kobe, Japan). The SII was calculated from the platelet counts (reference range: 100–300 × 10^9^/L), neutrophil counts (reference range: 4–10 × 10^9^/L), and lymphocyte counts (reference range: 1.1–3.2 × 10^9^/L), using the following formula: SII = platelet×neutrophil/lymphocyte counts as defined previously [28]. The SII was expressed as × 10^9^/L.

### Follow-up and outcome

After discharge, all living patients in the hip fracture database were telephonically followed up monthly for the first 3 months, and then every 3 months until the 1 year, and every 6 months thereafter. The follow-up contents included survival status (alive/death), time of death and cause of death. The date of in-hospital death were obtained from electronic medical records. Survival time was calculated from the date of hospital admission to either the date of death from any cause or last follow-up (December 31, 2019), whichever came first. The outcomes were all-cause mortality at 30 days, 1 year and last follow-up.

### Assessment of potential confounders

A number of variables were extracted from the hip fracture database, including demographic and clinical characteristics, and other laboratory data. Time to surgery was calculated as the date of surgery minus hospital admission date. The presence of comorbidity was evaluated using Charlson comorbidity index (CCI) score, which comprises 17 comorbid conditions and assigned a weigh of 1 to 6 points according to its impact on mortality [29]. Based on prior studies, CCI was categorized as none (CCI = 0), low (CCI = 1), or high (CCI ≥ 2) [4]. The reference range of albumin was 3.5–5.5 g/dL, and no patient exceeded the upper limit. The albumin levels were then classified into hypoalbuminemia (albumin < 3.5 g/dL) and normal albumin (albumin ≥3.5 g/dL) [30].

### Statistical analysis

All data received a double check before the analysis, and missing data were identified, including serum albumin (*n* = 7, 2.4%), time to surgery (*n* = 5, 1.7%). While the missing data were continuous variables, and the percentages were low, they were supplemented with simple imputations using the median nonmissing value, as described elsewhere [31]. Continous data were expressed as means (standard deviation, SD) or median (interquartile range, IQR) according to the distribution, and examined by independent Student’s *t*-test for normally distributed variables, and Wilcoxon rank-sum test for non-normally distributed variables. Categorical data were described as frequencies (percentages), and compared by *χ*^*2*^ test.

The median follow-up time was identified as a median observation time of patients who were still alive at last follow-up, and estimated by the reverse Kaplan-Meier method. Univariable Cox proportional hazard models were carried out to estimate the effect of each predictor on 30-day, 1-year and total mortality. Further multivariable Cox analyses were performed to identify the independent risk factors for survival. Proportional hazard assumption was assessed for all variables, using the Kaplan-Meier estimates for the categorical variables and the Schoenfeld’s residuals for the countinous variables [32]. Owing to the small number of death, we did not perform multivariable adjustment for 30-day mortality, and other multivariable models were adjusted for the following potential confounders identified in the previous literature [30, 33], including age, sex, time to surgery, CCI score and hypoalbuminemia. In these models, SII value was divided by 100 in order to improve the readability of hazard ratio (HR).

Moreover, subgroup analyses were performed according to age (< 80 or ≥ 80 years), sex (male or female), CCI score (0, 1, ≥ 2) and albumin level (≥ 3.5 g/dL or <  3.5 g/dL). We also conducted a test for the interaction between SII and each subgroup factors.

All statistical analyses were performed using JMP Pro software (version 13.0.0; SAS Institute Inc., Cary, NC, USA). A two-tailed *p* <  0.05 was considered statistically significant.

## Results

### Baseline characteristics

**Fig. 1:**
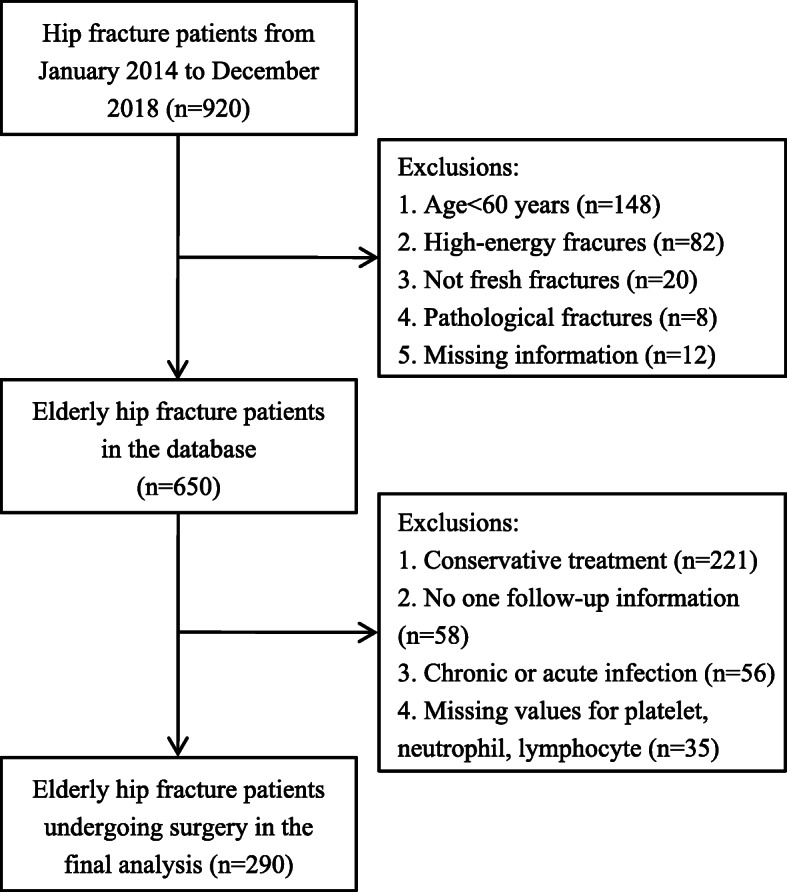
Flow chart of the study participants

Between January 2014 and December 2018, a total of 920 hip fracture patients treated at our orthopaedic center, and 660 patients have been consecutively included in the hip fracture database. In this study, we excluded those who received conservative treatment (*n* = 221), had no one follow-up information (*n* = 58), chronic or acute infection within 48 h of admission (*n* = 56), and missing values for platelet, neutrophil, lymphocyte counts (*n* = 35). Finally, 290 patients undergoing surgery were included in the final analysis (Fig. 1).

The baseline characteristics are presented in Table 1. The mean (SD) age was 77.6 (8.6) years, and 65.2% were female. According to the CCI score, 159 (54.8%) patients were classified as none comorbidity, 89 (30.7%) patients as low comorbidity, and 42 (14.5%) patients as high comorbidity. The most common type of hip fracture was femoral neck (54.5%), and more than half (55.9%) underwent internal fixation surgery, and the median (IQR) time to surgery was 5.6 (4.0–7.5) days, and 25.9% were hypoalbuminemia. Moreover, the median (IQR) SII was 759.4 (519.0–1128.7) × 10^9^/L.

**Table 1:** Baseline characteristics

Characteristics	Total (***N*** = 290)
Age (years), mean (SD)	77.6 (8.6)
Female, n (%)	189 (65.2)
CCI score, n (%)	
CCI = 0	159 (54.8)
CCI = 1	89 (30.7)
CCI ≥ 2	42 (14.5)
Type of hip fracture, n(%)	
Femoral neck	158 (54.5)
Intertrochanteric	132 (45.5)
Type of surgery, n (%)	
Internal fixation	162 (55.9)
Arthroplasty	128 (44.1)
Time to surgery (days), median (IQR)	5.6 (4.0–7.5)
Platelet (×10^9^/L), median (IQR)	139.0(102.0–177.3)
Neutrophil (×10^9^/L), median (IQR)	5.6 (4.4–7.4)
Lymphocyte (×10^9^/L), median (IQR)	1.0 (0.8–1.3)
SII (×10^9^/L), median (IQR)	759.4(519.0–1128.7)
Albumin (g/dL), mean (SD)	3.8 (0.4)
Hypoalbuminemia, n(%)	75 (25.9)

Continous data were expressed as mean (SD) or median (IQR) according to the distribution, and categorical data were described as frequencies (percentages)

*Abbreviation*: *SD* Standard deviation, *CCI* Charlson comorbidity index, *IQR* Interquartile range, *SII* Systemic immune-inflammation index

### Mortality

**Fig. 2:**
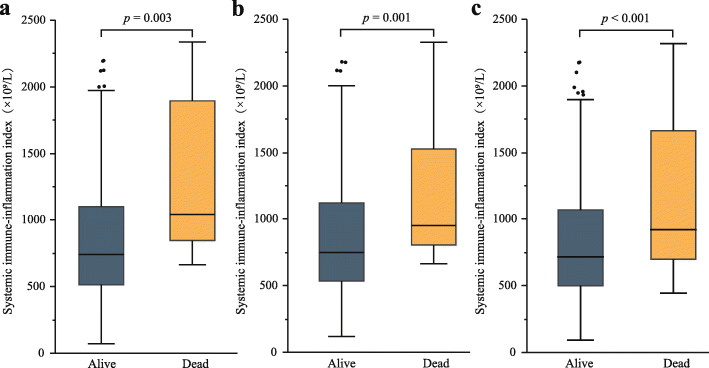
Box-plots comparing systemic immune-inflammation index (SII) among alive and dead patients at different times. **a** 30 days; **b** 1 year; **c** last time of follow-up. Blue box plots represent alive patients, whereas yellow box plots represent dead patients. The horizontal lines within the boxes represent the median; the boxes represent the interquartile range; the ends of the whiskers represent the minimum and maximum values, excluding the outliers; dots above and below the box plots represent outliers. Differences between groups were analyzed by Wilcoxon rank-sum test

After a median (IQR) follow-up time of 33.4 (22.8–48.9) months, 54 patients (18.6%) died, of which 3 deaths (1.0%) occurred in hospital. Overall, the mortality rates at 30 days and 1 year were 4.5% (*n* = 13) and 9.0% (*n* = 26), respectively. Compared with patients still alive, deceased patients had a significantly higher SII value at 30 days, 1 year and last follow-up (Fig. 2).

### Risk factors for predicting mortality

As shown in Table 2, univariable analysis showed that increasing age, male, high CCI score, time delay for surgery, hypoalbuminemia, and SII were associated with increased 30-day, 1-year and total mortality. Both types of hip fracture and surgery were not found to be risk factors. After adjusting for other covariates (Table 3), each increase of 100 units of SII was associated with a 8% increased hazard of death at 1-year follow-up (HR = 1.08, 95% CI: 1.01–1.17, *p* = 0.033), and 9% increased hazard of death at last follow-up (HR = 1.09, 95% CI: 1.03–1.15, *p* = 0.003). Moreover, male, high CCI score, and hypoalbuminemia were significantly associated with 1-year mortality, and increasing age, high CCI score, time delay for surgery, and hypoalbuminemia were significantly associated with total mortality.

**Table 2:** Univariable Cox regression analysis for the risk factors associated with 30-day, 6-month and 1-year mortality

Variables	30-day mortality		1-year mortality		Total mortality	
	HR (95% CI)	***p*** value	HR (95% CI)	***p*** value	HR (95% CI)	***p*** value
Age (per 1 year increase)	1.12 (1.04–1.21)	0.002	1.07 (1.02–1.12)	0.008	1.08 (1.04–1.12)	< 0.001
Sex (male vs. female)	4.40 (1.43–16.24)	0.009	3.81 (1.74–8.94)	0.001	2.11 (1.24–3.62)	0.007
CCI = 0	1.00 (Reference)	–	1.00 (Reference)	–	1.00 (Reference)	–
CCI = 1	1.79 (0.33–9.69)	0.478	1.82 (0.62–5.32)	0.266	1.35 (0.67–2.65)	0.396
CCI ≥ 2	9.66 (2.68–44.83)	0.001	7.53 (3.03–20.24)	< 0.001	5.03 (2.69–9.42)	< 0.001
Fracture type (intertrochanteric vs. neck)	1.97 (0.66–6.52)	0.226	1.23 (0.57–2.68)	0.595	1.62 (0.94–2.80)	0.083
Surgery type (fixation vs. arthroplasty)	1.81 (0.59–6.70)	0.306	1.10 (0.51–2.45)	0.816	1.15 (0.67–1.99)	0.604
Time to surgery (per 1 day increase)	1.10 (0.83–1.30)	0.411	1.10 (1.01–1.17)	0.023	1.14 (1.08–1.19)	< 0.001
Albumin (< 3.5 g/dL vs. ≥ 3.5 g/dL)	4.77 (1.59–15.79)	0.006	4.27 (1.97–9.53)	< 0.001	2.52 (1.46–4.32)	0.001
SII (per 100 units)	1.16 (1.05–1.28)	0.003	1.11 (1.04–1.19)	0.004	1.12 (1.07–1.18)	< 0.001

*Abbreviation*: *HR* Hazard ratio, *CI* Confidence interval, *CCI* Charlson comorbidity index, *SII* Systemic immune-inflammation index

**Table 3:** Multivariable Cox regression analysis for the risk factors associated with 1-year and total mortality

Variables	1-year mortality		Total mortality	
	HR (95% CI)	***p*** value	HR (95% CI)	***p*** value
Age (per 1 year increase)	1.04 (0.98–1.10)	0.173	1.06 (1.02–1.10)	0.003
Sex (male vs. female)	2.82 (1.26–6.71)	0.011	1.72 (0.98–3.01)	0.056
CCI = 0	1.00 (Reference)	–	1.00 (reference)	–
CCI = 1	1.35 (0.45–4.02)	0.585	1.00 (0.49–1.98)	0.991
CCI ≥ 2	5.04 (1.84–14.51)	0.002	2.97 (1.45–5.98)	0.003
Time to surgery (per 1 day increase)	1.02 (0.99–1.09)	0.639	1.07 (1.02–1.13)	0.016
Albumin (< 3.5 g/dL vs. ≥ 3.5 g/dL)	2.93 (1.29–6.88)	0.010	1.83 (1.03–3.22)	0.040
SII (per 100 units increase)^b^	1.08 (1.01–1.17)	0.033	1.09 (1.03–1.15)	0.003

Adjusted for age, sex, CCI score, time to surgery and albumin level

*Abbreviation*: *HR* Hazard ratio, *CI* Confidence interval, *CCI* Charlson comorbidity index, *SII* Systemic immune-inflammation index

### Subgroup analysis

To further verify whether the predictive role of SII was consistent among different clinical situations, we performed subgroup analyses (Table 4). Consistent with the overall results, SII was also significantly associated with poor 1-year and total mortality. Moreover, there was no significant interaction between all the subgroup factors.

**Table 4:** Subgroup analysis by age, sex, CCI score and albumin for 1-year and total mortality

Subgroups	1-year mortality			Total mortality		
	HR (95% CI)	***p*** value	Interaction ***p*** value	HR (95% CI)	***p*** value	Interaction ***p*** value
Age			0.693			0.632
< 80 years	1.10 (0.95–1.26)	0.171		1.09 (0.99–1.20)	0.093	
≥80 years	1.10 (1.00–1.21)	0.041		1.10 (1.03–1.18)	0.005	
Sex			0.357			0.833
Male	1.11 (1.01–1.21)	0.030		1.08 (1.01–1.16)	0.031	
Female	1.04 (0.89–1.19)	0.595		1.10 (1.01–1.19)	0.011	
CCI score			0.686			0.241
CCI = 0	1.06 (0.91–1.22)	0.425		1.13 (1.03–1.23)	0.009	
CCI = 1	1.13 (0.99–1.29)	0.073		1.12 (1.01–1.23)	0.027	
CCI ≥ 2	1.08 (0.95–1.23)	0.247		1.04 (0.94–1.16)	0.413	
Albumin			0.639			0.710
≥ 3.5 g/dL	1.12 (1.00–1.24)	0.034		1.09 (1.02–1.18)	0.019	
< 3.5 g/dL	1.11 (1.00–1.22)	0.051		1.08 (1.00–1.17)	0.063	

Adjusted for age, sex, CCI score, time to surgery and albumin level

*Abbreviation*: *HR* Hazard ratio, *CI* Confidence interval, *CCI* Charlson comorbidity index

## Discussion

In this study, the overall mortality at 30 days, 1 year and total mortality at last follow-up were 4.5, 9.0 and 18.6%, which were lower than that in other countries [5, 6]. A recent systematic analysis calculated the 1-year mortality rate after hip fracture in mainland China, and the estimated mortality was 13.96% (95% CI: 12.26–15.86%) [34]. A study from Singapore also showed that the mortality was lower, and the rate was 1.8% at 30 days, and 13.3% at 2 years [3]. In the present study, many of those patients (33.5%) were treated conservatively and had a higher mortality rate, but we excluded these patients in the final analysis due to different clinical features. This may be the reason for the lower mortality rates in our study.

Recently, the relationship between inflammation and prognosis has attracted more attention in older adults [35, 36]. Bermejo-Bescós et al. [14] found that peripheral IL-6 level was significantly associated with a higher risk of 1-year mortality after hip fracture in patients over 80 years. Several biochemical markers of inflammation, such as levels of CRP, the soluble urokinase plasminogen activating receptor (suPAR) and ferritin, were found to be associated with 30-day mortality after hip fracture [15]. Hip fracture also led to significant systemic inflammation and acute lung injury in rat models, and the older rats suffered a more remarkable acute lung injury after hip fracture than the younger rats [37]. Although other study has found no such relationship between high inflammatory markers and 2-year mortality in hip fracture patients [38], most studies suggest that systemic inflammation may be related to the poor survival of hip fracture.

The SII is an objective marker to reflect the systemic inflammation, and easy to calculate by the platelet×neutrophil/lymphocyte counts formula. In older adults with hip fracture, more than half of the patients (62.6%) had low lymphocyte counts [39]. A recent meta-analysis reported that low total lymphocyte counts were significantly associated with higher total mortality (HR = 1.67, 95% CI: 1.28–2.18) [40]. Although there is no direct evidence that increased neutrophil counts are related to poor prognosis, elevated neutrophil-to-lymphocyte ratio was found to be an independent risk factor for postoperative myocardial injury, in-hospital death and 1-year mortality after hip fracture surgery [18, 19]. On the other hand, high platelet counts were considered to be a risk factor for developing a postoperative pressure ulcer after hip fracture surgery [41], and elevated PLR was found to be associated with increased all-cause mortality, especially in the older adults [42]. Recently, we also reported that high PLR (≥ 189) was associated with increased 1-year all-cause mortality in older adults with hip fracture [27]. Therefore, we speculated that elevated SII level by raising platelet, neutrophil counts and/or lowering lymphocyte counts may be possible to predict poor prognosis in older adults with hip fracture.

As expected, we first observed that dead patients had a significantly higher SII value in comparison with survivors. Moreover, multivariable analysis confirmed that SII was significantly associated with 1-year mortality (HR = 1.08 per 100 units, 95% CI: 1.01–1.17), and total mortality at last follow-up (HR = 1.09 per 100 units, 95% CI: 1.03–1.15). To further verify whether the predictive role of SII was consistent among different clinical situations, subgroup analyses also revealed that SII remained as an independent risk factor for survival in older adults with hip fracture, and there was no significant interaction.

Furthermore, our study showed that male, high CCI score, and hypoalbuminemia were significantly associated with 1-year mortality, and increasing age, high CCI score, time delay for surgery, and hypoalbuminemia were significantly associated with total mortality. These risk factors have been proven in other studies [3–5, 30, 33]. In this study, increasing age and time delay for surgery lost significance as independent factors for 1-year mortality. This may be due to a small number of death within 1-year follow-up.

However, our study also had several limitations. First, the sample size was small, this reduced the statistical power of the study. Meanwhile, the small number of deaths limited the ability to interpret our data on short-term mortality. Further studies with larger sample sizes are required to confirm our findings. Second, we did not obtain some variables related to survival, such as body mass index [43], time from fracture to hospital admission [44]. These unadjusted potential confounding factors may influence the results of this study. Third, only laboratory data at admission were entered in the hip fracture database, this also limited us to explore the relationship between SII at other time points and survival after hip fracture.

## Conclusions

Our findings demonstrate that SII is significantly associated with poor all-cause mortality in older adults with hip fracture undergoing surgery, and it may be a good index to predict the prognosis. Because SII is a simple and economical biomarker, it can be easily performed in usual clinical practice.

## Data Availability

The datasets used and /or analyzed during the current study are available from the corresponding author on reasonable request.

## Abbreviations

SII: Systemic immune-inflammation index
HR: Hazard ratio
SD: Standard deviation
IQR: Interquartile range
NHFS: Nottingham Hip Fracture Score
POSSUM: Physiologic and Operative Severity Score for the enUmeration of Mortality and Morbidity
AHFS: Almelo Hip Fracture Score
CCI: Charlson Comorbidity Index
IL-6: Interleukin-6
CRP: C-reactive protein
PNI: Prognostic nutritional index ratio
NLR: Neutrophil-to-lymphocyte ratio
CI: Confidence interval
suPAR: The soluble urokinase plasminogen activating receptor
PLR: Platelet-to-lymphocyte ratio
